# Elastomeric Cellular Structure Enhanced by Compressible Liquid Filler

**DOI:** 10.1038/srep26694

**Published:** 2016-05-25

**Authors:** Yueting Sun, Xiaoqing Xu, Chengliang Xu, Yu Qiao, Yibing Li

**Affiliations:** 1 State Key Laboratory of Automotive Safety and Energy, Tsinghua University, Beijing, 100084, P.R. China; 2 Department of Structural Engineering, University of California—San Diego, La Jolla, CA 92093-0085, USA

## Abstract

Elastomeric cellular structures provide a promising solution for energy absorption. Their flexible and resilient nature is particularly relevant to protection of human bodies. Herein we develop an elastomeric cellular structure filled with nanoporous material functionalized (NMF) liquid. Due to the nanoscale infiltration in NMF liquid and its interaction with cell walls, the cellular structure has a much enhanced mechanical performance, in terms of loading capacity and energy absorption density. Moreover, it is validated that the structure is highly compressible and self-restoring. Its hyper-viscoelastic characteristics are elucidated.

Developing high-performance energy absorption materials and structures has been an active research area for centuries^1^. In principle, any phenomenon that involves dissipative volume reduction can be employed for energy absorption. Cellular structures (e.g., foams, honeycombs and lattices) are widely used due to their high compressibility, which helps extend the duration and lower the intensity of impact pulses^2^,^3^,^4^. With the excellent conforming and self-restoring characteristics^5^,^6^, elastomeric cellular structures are particularly attractive for human body protection, as the confinement on human motion is secondary. A major bottleneck is the relatively low energy absorption capacity. In many cases, especially for intense nonlinear stress waves, elastomeric cellular structures fail to meet the increasingly high requirements.

A typical method to enhance cellular structures is to raise their stiffness and strength^7^, by adding fillers to render the system two-phased^8^. Solid fillers are commonly used. However, they may significantly reduce the flexibility of cell walls, and the processing is sophisticated. In the current study, we investigated liquid fillers. Because regular liquids are incompressible and have little energy absorption ability, we employed a novel liquid-like material, NMF liquid.

A NMF liquid is a liquid suspension of nanoporous particles. Our previous experimental research^9^,^10^,^11^,^12^ showed that NMF liquids are highly compressible, because the liquid can be intruded into the hydrophobic nanopores once the pressure exceeds a critical value, known as the infiltration pressure *P*_in_; that is, the volume of NMF liquid can be largely reduced upon a compressive loading. Under ambient condition, the liquid phase would remain outside the nanopores, due to the strong nanometer-scale capillary effect. Associated with the liquid infiltration process, a certain amount of mechanical energy, *P*_in_⋅*V*_p_, is dissipated into thermal energy and nanoscale interfacial energy, with *V*_p_ being the nanopore volume. As unloading takes place, the intruded liquid may or may not stay in the nanopores, for which the detailed mechanism is still relatively unclear.

In the past, NMF liquids were contained in rigid cells^13^,^14^,^15^; herein, a polyurethane cellular structure filled with NMF liquid was fabricated and thoroughly investigated. The results suggested that the NMF liquid filler can considerably enhance the loading capacity and the energy absorption density of this flexible cellular structure, and more importantly, the filled structure remains to be highly compressible. Such a liquid-containing composite structure has high flexibility, compressibility, recoverability, and energy absorption capacity, and therefore, offers technological advantages for vibration damping, body protection, etc.

## Results

**Figure 1:**
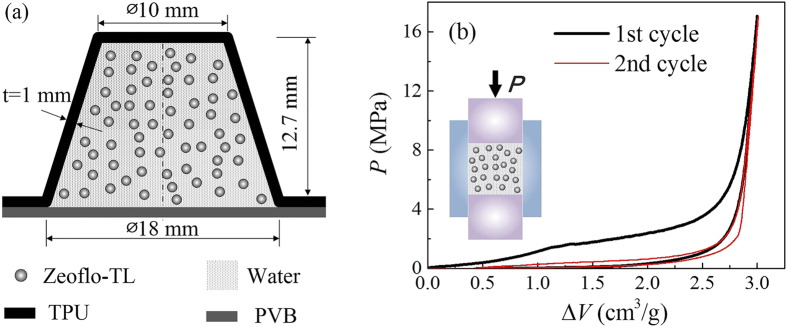
Testing sample. (**a**) NMF liquid filled TPU cell. (**b**) *P*-Δ*V* curves of Zeoflo-TL/water system, attached with its testing setup.

Figure 1a shows a typical cell of the polyurethane structure. It’s in the shape of truncated cone and similar to the hemi-ellipsoidal structure of SKYDEX pad, which proves to be stable and compatible to dynamic impact^16^. This structure is produced by a thermoplastic polyurethane (TPU) elastomer from BASF, Elastollan 1185A, via injection molding and precision machining. Then the cell is filled with a NMF liquid consisting of 1.2 g deionized water and 0.3 g Zeoflo-TL reversed phase nanoporous silica gel (Huber Corp.), and sealed by a layer of PVB (polyvinyl butyral) film using cyanoacrylate adhesive. Zeoflo-TL is a kind of precipitated silica, which is hydrophobic as received. So it provides an infiltration pressure *P*_in_ = 1.6 MPa, and nanopore volume *V*_p_ = 1.5 cm^3^/g, estimated from the quasi-static *P*-Δ*V* curve (pressure-specific volume change curves) in Fig. 1b. That means water molecules cannot enter its nanopores until the pressure reaches 1.6 MPa. When infiltration starts, a plateau of 1.5 cm^3^/g is formed, which then ends with a sharp reduction of liquid compressibility, once all nanopores are filled up with water molecules. The recompression on this NMF liquid exhibits no infiltration plateau, indicating non-outflow of water molecules during the unloading process. The energy absorption density of this NMF liquid is calculated to be 4.5 J/g by the hysteresis area between the loading and unloading curve.

**Figure 2:**
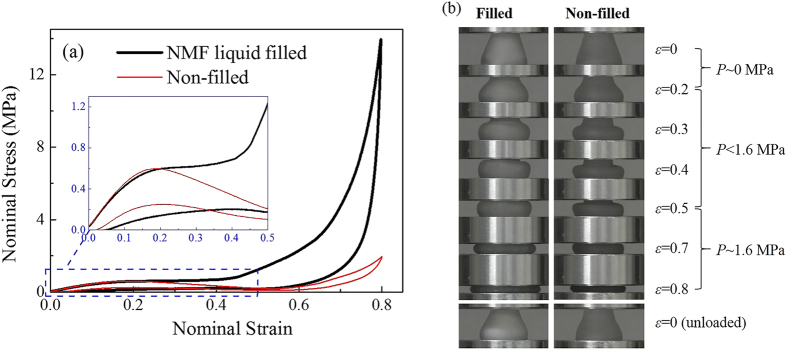
Compression test results of NMF liquid filled cell and non-filled cell under quasi-static condition. (**a**) Stress-strain curves. (**b**) Cell configurations.

The compressive loading and energy absorption performance of this NMF liquid filled TPU cell is experimentally studied. Figure 2a shows its nominal stress-strain curve under quasi-static condition (5 mm/min), along with the result of its non-filled counterpart. Upon compression, both cells show a linear elastic behavior first with increasing stress. Around strain 0.2, two curves bifurcate, with a stress decrement for the non-filled cell, but not for the NMF liquid filled cell. Then both structures will have a densification stage starting from larger strains around 0.5, characterized by a sharp growth of stress. Upon unloading, the NMF liquid filled cell experiences a drastic falling of compressive stress, quickly down to the same level of the non-filled cell. Then both of them keep a stress above zero until they’re unloaded to a strain less than 0.05. Figure 2b provides the buckling shapes of these two cells during the loading-unloading process. It’s shown that their differences mainly lie in the post buckling stage (*ε* > 0.2), and the initial buckling stage (*ε* = 0–0.2) exhibits trivial distinction. Both of them recover their shapes when completely unloaded. These are coherent with the findings from their stress-strain curves.

NMF liquid filler can substantially enhance the loading and energy absorption capacity of these cellular structures, indicated by the compressive stress level and hysteresis area in Fig. 2a. The energy absorption of the non-filled cell is merely contributed by the viscoelasticity of TPU material and elastic instability of structure, while for the NMF liquid filled cell, its NMF liquid filler will absorb considerable amount of energy through its nanoscale infiltration mechanism, and besides, its cell wall material will also experience a larger deformation and energy absorption due to its interaction with the liquid filler. The NMF liquid filled cell will therefore obtain a higher energy absorption capacity. However, compared with capacity, density is more worthy of concern for energy absorption. One optimistic finding is that either in terms of weight or volume, energy absorption density of the cell is significantly increased by adding NMF liquid filler. As shown in Table 1, aside from a 286% increase in the average loading stress, the energy absorption density is also highlighted by an increase of 157% in J/g and 423% in J/cm^3^. This should be attributed to the light weight and energy absorption ability of NMF liquid, so that its filling contributes more to the energy absorption instead of mass weight.

Generally, the compressibility of cellular structures will be weakened after adding fillers, while the NMF liquid filled structure here can achieve a compressive strain of 0.8 without any plastic failure or leakage. This is also nearly the maximum strain for the non-filled cell, when the cell wall is contacted and the whole structure becomes densified. Thus it can be inferred that adding NMF liquid filler is an effective way to enhance cellular structures with almost no loss in compressibility. This should be attributed to the compressibility and fluidity of NMF liquid filler. On one hand, NMF liquid is highly compressible, so the cell wall bears a moderate level of inner pressure, with no threat of failure throughout the loading process. On the other hand, NMF liquid can better accommodate the cell wall crushing compared with conventional solid fillers, by flowing laterally and increasing its effective sectional area. Thereby, under same porosity, NMF liquid filler can contribute to a higher compressibility.

As a major advantage of elastomeric cellular structures, recoverability should also be evaluated after the filling of NMF liquid. The unloading curves and final configurations in Fig. 2 have demonstrated that the NMF liquid filled cell is self-restoring, exhibiting a nearly complete recovery with trivial permanent deformation (<5%). This is also contributed by the liquid nature of the filler, which makes it more flexible than the elastomeric cell wall and provides almost no resistance to its shape recovery. Nonetheless, it should be noted that due to the cyclic softening effect of TPU material^17^ and the non-outflow property of Zeoflo-TL/water system, recompression on the NMF liquid filled cell will not completely repeat the first loading in response, typically characterized by a lower compressive stress and smaller hysteresis. If consistent performance is pursued, recoverable NMF liquids (e.g., the combination of Fluka 100-C_8_ silica gel and saturated sodium chloride^18^) can be adopted.

## Discussion

**Figure 3:**
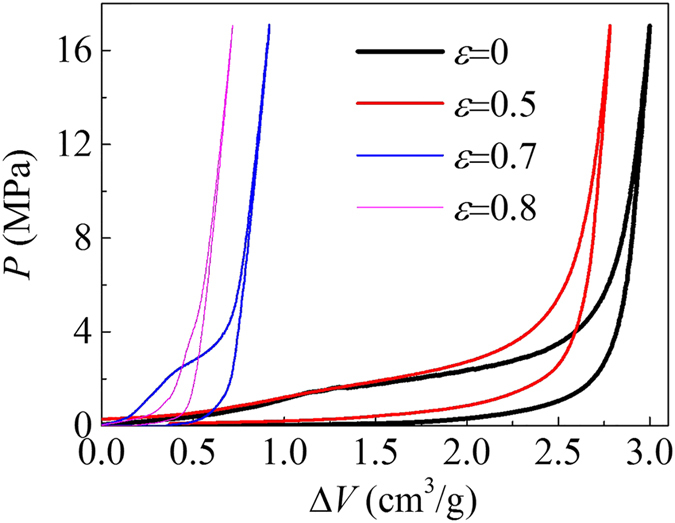
*P*-Δ*V* curves of the NMF liquid after cell compression tests with different strains.

The above results demonstrate a highly compressible and recoverable energy absorption cellular structure by combining the merits of elastomer and NMF liquid. In order to reveal what happens on the NMF liquid inside the cell during its compression, NMF liquid is taken out of the cell at each strains to have an infiltration test. Due to its non-outflow property, the available pore volume detected in the infiltration test are exactly those not occupied during the cell compression. Therefore, the infiltration test results can disclose the liquid filler’s infiltration and energy dissipation history during the cell compression. Figure 3 shows the *P*-Δ*V* curves of NMF liquid filler after a cell compression up to strains of 0.5, 0.7 and 0.8, respectively. The infiltration plateau of NMF liquid after a cell compression of strain 0.5 is nearly identical to that without cell compression (*ε* = 0). This indicates that tiny amount of pore volume has been occupied during the strain range *ε* = 0–0.5, i.e., the NMF liquid infiltration does not start until the cell is compressed to a strain around 0.5. The *P*-Δ*V* curve for *ε* = 0.7 still exhibits a short plateau, while for *ε* = 0.8, the curve is almost linear without plateau, suggesting that the NMF liquid infiltration does not end until the cell is compressed to a strain of 0.8. Therefore, the infiltration of NMF liquid filler predominantly happens within the strain 0.5–0.8 of the cell compression.

The above observation will help further understand the testing results in Fig. 2. In the initial buckling stage (*ε* = 0–0.2), the cell has almost no shrinkage of internal volume due to its lateral expansion, so the internal pressure from the NMF liquid filler is negligible. The NMF liquid filled cell therefore presents a similar stress-strain behavior and shape configuration with the non-filled cell in this stage. At larger strains (the post buckling stage *ε* > 0.2), the cell volume significantly decreases, and thereby a considerable pressure is imposed on the cell wall. This internal pressure suppresses the cell wall buckling, resulting in a higher compressive stress and a different configuration compared with the non-filled cell, as shown in Fig. 2. But the internal liquid pressure does not reach 1.6 MPa (*P*_in_) until *ε* = 0.5, when the infiltration starts and keeps the liquid pressure at 1.6 MPa until the end of compression. During the infiltration process, the liquid volume is significantly reduced and the mechanical energy is dissipated, through the intrusion of water molecules into nanopores. Since all nanopores are occupied at the end of compression, the energy absorbed by NMF liquid can be estimated to be 1.35 J (by multiplying 0.3 g with 4.5 J/g). The rest of the energy 2.42 J (3.77 J in total) is absorbed by the cell wall, which is also significantly higher than that of the non-filled cell 0.72 J.

**Figure 4:**
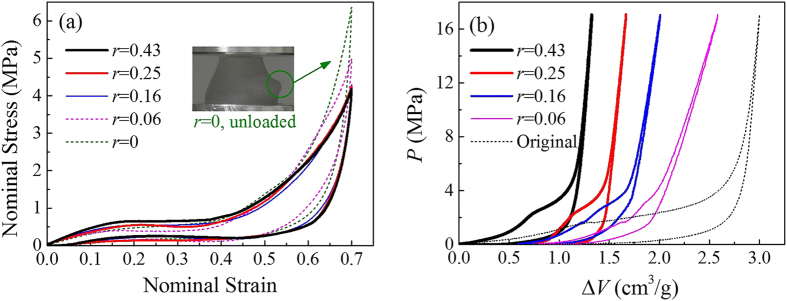
Compression test results under various mass ratios of Zeoflo-TL silica gel to deionized water. (*r* = 0 corresponds to 2.20 g water without silica; *r* = 0.06 corresponds to 0.10 g silica and 1.60 g water; *r* = 0.16 corresponds to 0.20 g silica and 1.27 g water; *r* = 0.25 corresponds to 0.30 g silica and 1.18 g water; *r* = 0.43 corresponds to 0.40 g silica and 0.93 g water). (**a**) Stress-strain curves, attached with a photo of cell wall failure (*r* = 0, unloaded). (**b**) *P*-Δ*V* curves of the NMF liquid after cell compression tests, along with the original curve without cell compression for comparison.

It’s worth mentioning that the compressibility of NMF liquid depends on its mass ratio of nanoporous material to water *r*. Thus, an adequate mass ratio should be selected to ensure the NMF liquid filler to be compressible enough. Otherwise, at a certain cell compression strain when the nanopore volume is totally filled by water molecules, the sharply increased liquid pressure may lead to a failure of the cell wall by local plastic yielding. As shown in Fig. 4, the nanopore volume becomes insufficient at a cell compression of strain 0.7 if *r* < 0.16 (0.2 g silica with 1.27 g water), and it leads to an exceptionally high internal pressure and structure failure (an exceeding unrecoverable expansion and thinning of the cell wall with high threat of leakage), which means that the compressibility of the cell is cut down. Therefore, keeping a high mass ratio so as to maintain the liquid pressure around *P*_in_ throughout the loading process is recommended. In those circumstances, NMF liquid filled cells will have similar mechanical responses for different mass ratios, evidenced by the nearly identical curves in Fig. 4a when *r* > 0.16. However, a closer observation shows a slight increase of hysteresis and decrease of peak nominal stress with the mass ratio, which may be caused by the pore size distribution of silica (larger nanopores will be infiltrated first at a relatively lower pressure) and distinct modulus of NMF liquids with different mass ratios. Furthermore, a higher mass ratio means a lower mass density of NMF liquid since nanoporous material is lightweight. With these two effects combined together, the energy absorption density of NMF liquid filled cell will increase with its mass ratio. (Here, with *r* from 0.16 to 0.43, the energy absorption density at strain 0.7 increases from 0.55 J/g to 0.67 J/g). Therefore, a higher mass ratio is beneficial in terms of either compressibility or energy absorption density.

In summary, the experimental results demonstrate that the filling of NMF liquid can significantly enhance the mechanical performances of elastomeric cellular structures. Their loading capacity and energy absorption density can be substantially increased, meanwhile with satisfying compressibility and recoverability. This is attributed to the energy dissipation mechanism of NMF liquid by nanoscale infiltration, as well as its lightweight compressible liquid nature. These findings suggest that NMF liquid is an ideal filler material for flexible and resilient cellular structures, which may promote development of smart protection systems like body armors.

## Methods

### Sample preparation

The TPU material Elastollan 1185A was from BASF, received as transparent pellets. By injection molding, they were produced into bars with a diameter of 25 mm and machined into the truncated cone configuration. The cell shown in Fig. 1a has a height of 12.7 mm, a diameter of 10 mm at the flat top and a diameter of 18 mm at the bottom, and the wall thickness is 1 mm. For mass production of cellular structures, TPU pellets can be directly molded into designed shapes. The NMF liquid here was composed of Zeoflo-TL reversed phase silica gel (Huber Corp.) and deionized water. The Zeoflo-TL silica powder was firstly pre-compressed in a steel chamber with 2.5 MPa to effectively eliminate gaps. Then the specified amount of silica and deionized water were filled into the cell, and a PVB film with cyanoacrylate adhesive was utilized for sealing. Other polymers compatible with TPU could also be adopted. A stainless steel loading plate was used to fix the sample and further enhance the sealing effect.

### Compression tests

Compression tests on cell specimens were conducted using Instron 8872, which continuously measured the applied load *F* and displacement *d*. The loading and unloading rates were set to be 5 mm/min, regarded as a quasi-static condition. In the stress-strain curves, the nominal stress is defined as *σ* = *F*/*A*_b_, and the nominal strain is defined as *ε* = *d*/*h*, with *A*_b_ being area of the base and *h* being the height. For comparison purpose, NMF liquid filled cells and non-filled cells were tested under same conditions. The compression process was recorded by video camera.

### Infiltration tests

Pressure-induced infiltration tests on NMF liquids were carried out to identify the nanoporous parameters of Zeoflo-TL silica (Fig. 1b) and determine the infiltration history of NMF liquid filler during the cell compression tests (Figs 3 and 4b). The infiltration tests were conducted on Instron 8872, through the compression on NMF liquid sealed in a stainless steel chamber (shown in Fig. 1b) with precisely-fit sealing rings. The testing results for Fig. 1b involved 0.3 g silica with a pre-compression of 2.5 MPa, while for Figs 3 and 4b, all the silica inside the cell after compression was moved into the chamber. Then deionized water was added to reach an initial total volume of 5.3 ml. Due to its hydrophobic nature, Zeoflo-TL silica powder would not homogeneously distributed in water. The piston with a diameter of 19.1 mm was compressed into the chamber at 5 mm/min, creating a quasi-static loading pressure on NMF liquid. Once the pressure reached 17 MPa (5 kN), the piston moved back at the same velocity. In the *P*-Δ*V* curves, the pressure *P* is defined as *P* = *F/A,* with *A* being the cross-sectional area of the piston, and the specific volume change Δ*V* is defined as Δ*V* = (*A*·*d*)/*m*, with *m* being the mass of silica. The infiltration pressure *P*_in_ is taken as the pressure at the middle point of the transition zone, which equals 1.6 MPa here. The width of infiltration plateau suggests the pore volume available for liquid infiltration.

## Additional Information

**How to cite this article**: Sun, Y. *et al*. Elastomeric Cellular Structure Enhanced by Compressible Liquid Filler. *Sci. Rep.* **6**, 26694; doi: 10.1038/srep26694 (2016).

## Figures and Tables

**Table 1:** Mechanical performance of NMF liquid filled cell and non-filled cell.

**Filler**	**Average Stress (MPa)**	**Energy absorption density (J/g)**	**Energy absorption densify (J/cm**^3^)
Non-filled	0.51	0.51	0.20
NMF liquid	1.97	1.31	1.04
